# Influence of Facial Morphology on Masticatory Function and Quality of Life in Elders Using Mandibular Overdentures: 3-Year Results

**DOI:** 10.3389/fnut.2021.608095

**Published:** 2021-02-12

**Authors:** Anna Paula da Rosa Possebon, Alessandra Julie Schuster, Raissa Micaella Marcello-Machado, Ana Paula Pinto Martins, Luciana de Rezende Pinto, Otacílio Luiz Chagas-Júnior, Altair Antoninha Del Bel Cury, Fernanda Faot

**Affiliations:** ^1^Graduate Program in Dentistry, School of Dentistry, Federal University of Pelotas, Pelotas, Brazil; ^2^Department of Prosthodontics and Periodontology, Piracicaba Dental School, State University of Campinas, Piracicaba, Brazil; ^3^Department of Restorative Dentistry, School of Dentistry, Federal University of Pelotas, Pelotas, Brazil; ^4^Department of Oral and Maxillofacial Surgery and Maxillofacial Prosthodontics, School of Dentistry, Federal University of Pelotas, Pelotas, Brazil

**Keywords:** mandibular overdentures, facial profile, oral health-related quality of life, facial morphology, anteroposterior skeletal discrepancy, masticatory function

## Abstract

**Background:** Facial types may interfere in the oral health-related quality of life (OHRQoL) and masticatory performance of implant-retained mandibular overdenture (IMO) wearers.

**Purpose:** Investigate the medium-term changes in the masticatory function (MF) and OHRQoL parameters of IMO users, as a function of facial pattern, anteroposterior skeletal discrepancy, and sex.

**Methods:** Forty IMO users, most of them Caucasian (90%) with average age of 69.17 years were classified according to their facial pattern and antero-posterior discrepancy prior to rehabilitation. MF was evaluated by the multiple sieves method to determine the average particle size (X50), heterogeneity (B) and masticatory efficiency (ME, calculated as the percentage of material retained in the 5.6 and 2.8 mm sieves), using Masticatory performance (MP) and swallowing threshold (ST) tests. OHRQoL was measured by applying the dental impact on daily life (DIDL) questionnaire. The data were analyzed by Wilcoxon-paired tests to analyze changes in MF parameters over time, and mixed-effect multilevel regression models were employed to verify differences between groups.

**Results:** Significant changes were still observed in the 3rd year for the ST test with improvements in B for Mesofacial and in time for Dolichofacial individuals, while ME_2.8 deteriorated for Brachyfacial participants. B values of Class I and male individuals improved and brachyfacial individuals still presented worse homogenization (B) than Mesofacial participants in both masticatory tests. Class II and III participants still showed improvements in ME_5.6 and time compared to Class I despite increases in X50. Class II individuals needed less cycles than Class I in the 3rd year. Brachyfacial participants scored lower in the Appearance domain than Mesofacial ones in the 3rd year. Dolichofacial participants and Class III patients scored lower in the Oral Comfort domain than Mesofacial and Class I, respectively. In addition, age influenced the Pain, Oral Comfort and General Performance domains in the 3rd year.

**Conclusions:** Differences in facial morphology continue to influence the MF and OHRQoL outcomes in the 3rd year, and age influenced some OHRQoL domains. Brachyfacial individuals continue to benefit least from rehabilitation with IMO according to masticatory parameters.

## Introduction

The masticatory function (MF) of total edentulous individuals can be directly affected by the facial pattern (FP), by the anteroposterior discrepancy (ASD), and the type of prosthetic rehabilitation (1). According to the specialized literature, facial morphology shows cephalometric differences between ethnic groups (2–4). As an example, studies have shown that black and female individuals have a greater depth of the maxilla, whereas white individuals and men have a greater tendency for cranial deflection. Meanwhile, angular cephalometric measurements show no difference between these groups. The facial type is genetically established, and gender influences facial type, mainly through muscle pattern (3, 4). In terms of FP, Mesofacial individuals present a balanced bone profile and facial musculature, thus having a more predictable prognosis during prosthetic rehabilitation with conventional complete dentures (CCD), and consequently, they are considered the standard for comparisons (5, 6). Meanwhile, Brachyfacial individuals may present a greater bite force due to the biomechanical changes resulting from a compressed lower third of their face combined with strong muscle activity, which can contribute to a greater displacement of CCD during function (7, 8). Finally, Dolichofacial individuals present a greater bone height of the residual ridges in both jaws than Brachyfacial individuals (9), which would contribute to a greater stability of this type of rehabilitation with CCD and consequently a superior masticatory performance is expected. Thus, the bone and muscle characteristics of the different FP must be taken into account when planning treatment with complete dentures, to ensure a good prognosis of prosthetic rehabilitation in addition to ensuring the quality of MF (8, 10).

Another important factor capable of influencing the prognosis of totally edentulous rehabilitation is the anteroposterior discrepancy. In this framework, Class I individuals have balanced horizontal bone growth, resulting in have more predictable prosthetic rehabilitation than for Class II and Class III individuals, and thus are considered the standard for comparison (8, 10, 11). The mandibular protrusion of Class III individuals may result in a decreased vertical dimension of occlusion (VDO) (8), while the greater height of the residual bone crest in Class II individuals may enlarge VDO, which can inhibit reestablishment of the adequate maxillomandibular relationship (1, 7). The ASD deviations can often be compensated during prosthetic rehabilitation to improve MF. However, a previous study (9) found that Class III individuals had a reduced capacity to homogenize the bolus even after rehabilitation with new CCDs, suggesting that reestablishment of an effective masticatory pattern is extremely challenging in patients with this profile, and that they need a longer period to adapt to the prostheses.

Rehabilitation with implant mandibular overdentures (IMO) is preferable to the use of CCD, especially when patients experience difficulties adapting to CCD (12). IMO increase the retention and stability of the prostheses, and improve the comfort, speech and masticatory function of individuals, generating greater satisfaction with the treatment and an increase in self-reported oral health-related quality of life (OHRQoL) in the first 3 months of use (13). However, studies have shown that FP and ASD can affect the masticatory pattern that individuals develop even after the transition from CCD to IMO (1, 10, 11). Brachyfacial patients showed only a small short-term improvement in the test food trituration (10) and the FP no longer influenced the quality of chewing already after 1 year of IMO usage (11). Meanwhile, ASD negatively influenced the masticatory function, since Class II patients continued to present difficulties homogenizing the food compared to Class I individuals, both short term (10) and after 1 year of IMO use (11).

Presently, little is known about the medium-term influence of the facial morphology on OHRQoL of IMO users. In a clinical study with a 3-month follow-up time, the authors found that Dolichofacial individuals reported better scores in the Appearance and General Performance domains than Mesofacial individuals, while Class II Individuals reported higher Oral Comfort scores than Class I individuals (10). Despite the observed OHRQoL improvements of these individuals, the benefits of using IMO are perceived differently by the individuals in the short term. Thus, given the gap in the literature regarding the impact of factors such as facial pattern and the anteroposterior discrepancy may still have on MF and OHRQoL of IMO users, the objective of the present study was to investigate the medium-term changes in MF and OHRQoL parameters of IMO wearers as a function of FP, ASD, and sex. The null hypothesis of the study is that these parameters do not vary over time and that differences in the aspects PF, ASD, sex, and age are not able to influence MF and OHRQoL in the 3rd year of function.

## Materials and Methods

This longitudinal clinical study reports 3 year follow-up results of a previous study (10) performed with totally edentulous individuals assessed before transition from CCD to IMO and after 3 months. Initially, all volunteers were rehabilitated with new CCD, which were made with thermo-polymerizable acrylic resin (VIPICRIL plus - VIPI®), artificial acrylic resin teeth (Trilux - VIPI®) assembled in bilateral occlusion. The new protheses were fabricated in the Complete Denture Clinic by undergraduate students under the supervision of two specialized professors (FF, LRP). Panoramic and lateral radiographs for all participants were performed on a Rotograph Apparatus Plus instrument by a single trained and calibrated technician. The facial pattern (FP) and anteroposterior skeletal discrepancy (ASD) classifications were performed with cephalometric analysis software (CefX version 4.5.10), using cephalometric tracings as described in the previous clinical study (9, 10). Thus, individuals were classified as Mesofacial, Brachyfacial or Dolichofacial through Rickets' analysis, based on 5 angles (14). The ASD classification into class I, II or III was based on 3 angles (15).

The original study recruited completely edentulous elderly participants of both sexes with good general and oral health according to the following inclusion criteria: users of conventional complete dentures with difficulties adapting to a mandibular complete denture, adequate oral hygiene, without self-reported systemic impairments, and with bone heights ≥ 10 mm in the anterior region of the mandible. Participants presenting serious systemic diseases that compromised bone healing were excluded, along with uncontrolled diabetes, history of radiotherapy in the head or neck region, previous history of oral implants installation, and participants who underwent treatment with bisphosphonates in the preceding 12 months. At the moment of the 3-year follow-up visit, all participants were ≥65 years and all prostheses were of good quality [category 0 according to the criteria of Vigild (16)].

After 3 months of the adaptation to the new CCD, two narrow diameter implants (Facility implant: 2.9 × 10 mm; Ti grade V, NeoPoros surface - Neodent®) were installed in the region between mental foramina and immediately connected to healing caps. After a 3-month osseointegration period, the healing caps were replaced by Equator attachments, and the IMO were installed. All implant surgeries were performed by a specialized surgeon (OLCJ) and the IMO were made by prosthodontists. In the previous short-term study, 56 individuals were evaluated; 42 of them (29 women and 13 men) met the inclusion criteria, signed the informed consent form, and participated in the study. Volunteers who presented decompensated diabetes, uncontrolled hypertension, hemorrhagic disorders, severe systemic diseases, compromised immune system, or a history of radiotherapy in the head or neck region were excluded. The participants in the aforementioned study had an average age of 66.31 years, an average time since mandibular edentulism of 24.14 years. Most individuals are Caucasian/white (90%), 1 is of Asian origin (2.5%) and 3 are brown/black (7.5%). The sample comprised 33% Dolichofacial (8 women and 6 men), 31% Brachyfacial (9 women and 4 men), and 36% Mesofacial participants (12 women and 3 men). In terms of ASD, the sample consisted of 26% of Class I (6 women and 5 men), 29% Class II (7 women and 5 men) and 45% Class III participants (16 women and 3 men). This report follows the STROBE guidelines (17), was conducted in accordance with the Declaration of Helsinki 2008, and was approved by the Research Ethics Committee of the Faculty of Dentistry UFPel, protocol (No. 69/2013). The 42 volunteers were contacted via telephone for annual assessments 1–3 years after occlusal IMO loading for evaluation of masticatory function and oral health-related quality of life (OHRQoL) would be carried out.

To assess masticatory performance (MP), individuals were instructed to chew 17 cubes with sides of 5.6 mm (≈3.7 g) of “Optocal” test material for 40 cycles (18). During the swallowing threshold (ST) test, participants chewed another 17 cubes until they felt like swallowing, and the number of cycles and the time to execute the cycles were recorded (19). After both tests, the crushed material was expelled on a paper filter, dried at room temperature for 7 days and passed through multiple sieves. The material retained in each sieve was then weighed, and the average sieve opening through which 50% of the masticated material would pass (X50) and the homogeneity of the chewed particle distribution (B) were calculated. The masticatory efficiency parameters (ME_5.6 and ME_2.8) were calculated as the percentage of material retained in the 5.6 and 2.8 mm sieves (20, 21).

The OHRQoL was assessed through the DIDL questionnaire that assesses self-reported satisfaction through 36 questions divided into 5 domains: Appearance, Pain, Oral Comfort, General Performance, and Chewing (22, 23). The possible answers are agreed, neutral or disagreed, scored as +1, 0, and −1, respectively. All annual evaluations were performed by a single evaluator. Multilevel mixed effect regression models were used to estimate the effect of time on masticatory outcomes (MP, ST, and ME) and OHRQoL according to FP, ASD, sex and age, using Mesofacial and Class I patients as the reference groups. Regression coefficients and 95% confidence intervals were estimated, and *p*-values ≤ 0.05 were considered statistically significant. Intra-group changes in the masticatory parameters between the evaluation periods, as indicated by a significant time effect in the regression analysis, were assessed through the Wilcoxon-paired test using Bonferroni correction of the *P*-values (*P*-value required for significance = 0.05/3 = 0.0166). For the OHRQoL analyses, the effect size (ES) was calculated as the difference in the mean scores of the DIDL domains divided by the standard deviation of the previous evaluation period. The effect size was classified as small (ES < 0.5), moderate (0.5 < ES < 0.8) or large (ES ≥ 0.8) (24). All analyses were performed using the Stata 14.1 software (StataCorp).

## Results

Of the 42 individuals included in the initial study, 40 returned for evaluation at 1 and 3 years. The two follow-up losses were 2 women (1 Brachyfacial and Class III, and 1 Dolichofacial and Class I) and occurred due to loss of contact between 3 months and 1 year. The average age of the individuals evaluated in this period was 69.17 ± 3.93 years.

The mixed-effect multilevel regression models showed significant differences in B values between Brachyfacial and Mesofacial individuals in the 3rd year, both in the MP (*p* ≤ 0.01, Table 1) and in the ST test (*p* ≤ 0.01, Table 2). Brachyfacial individuals showed worse food homogenization, as indicated by B values that are 28.78% higher in the ST test and 39.23% higher the MP test. The ST outcomes of Class II and Class III individuals also differed from those of Class I individuals in the third year, with X50 values that were 3.03 and 13.37% higher for Class II and Class III individuals, respectively (*p* ≤ 0.01; *p* ≤ 0.01, respectively), and 48 and 2.49% higher ME_5.6 values for Class II and Class III individuals (*p* = 0.04; *p* = 0.03, respectively). In addition, the cycle time in Class II and Class III individuals was also 14.74 and 2.47% lower than for Class I individuals (*p* = 0.02; *p* = 0.04, respectively). Meanwhile, significant differences in the number of cycles were only found between Class II and Class I individuals (*p* ≤ 0.01), with a 6.09% reduction in the number of cycles at the end of the 3rd year.

**Table 1:** Mixed-effects regression model of the masticatory performance outcomes (MP- 40 cycles) according to facial pattern (FP), anteroposterior skeletal discrepancy (ASD), sex, and age.

**Masticatory performance test**				
**Outcomes** ** Time**	**MP_X50, coefficient (95%CI)**	**MP_B, coefficient (95% CI)**	**MP_ME _5.6, coefficient (95% CI)**	**MP_ME_ 2.8, coefficient (95% CI)**
3 months	Ref.	Ref.	Ref.	Ref.
1 year	**0.51 (0.14; 0.89)**	0.55 (−0.18; 1.28)	0.28 (−0.11; 0.69)	**0.46 (0.16; 0.76)**
3 years	0.27 (−0.07; 0.62)	0.36 (−0.34; 1.07)	**0.53 (0.12; 0.94)**	0.31 (−0.04; 0.67)
1–3 years	**0.58 (0.33; 0.82)**	**0.65 (0.42; 0.89)**	**0.66 (0.40; 0.92)**	**0.58 (0.22; 0.93)**
**FP**				
Mesofacial	Ref.	Ref.	Ref.	Ref.
Brachyfacial	0.58 (−0.72; 1.88)	**0.25 (0.04; 0.45)**	0.49 (−0.24; 1.22)	0.60 (−0.27; 1.48)
Dolichofacial	0.45 (−0.84; 1.79)	0.28 (−0.95; 1.52)	1.06 (−0.61; 2.73)	0.93 (−0.74; 2.60)
**ASD**				
Class I	Ref.	Ref.	Ref.	Ref.
Class II	0.80 (−1.10; 2.70)	0.02 (−2.02; 2.08)	1.44 (−3.04; 5.94)	1.42 (−0.75; 3.60)
Class III	0.11 (−0.79; 1.01)	−0.15 (−0.59; 0.27)	−0.00 (−1.30; 1.29)	−0.26 (−0.97; 0.44)
**Sex**				
Male	Ref.	Ref.	Ref.	Ref.
Female	0.15 (−0.29; 0.61)	−0.17 (−0.43; 0.07)	0.01 (−0.29; 0.31)	0.08 (−0.47; 0.65)
**Age (years)**	−0.01 (−0.05; 0.04)	−0.01 (−0.09; 0.06)	0.22 (−0.74; 1.19)	0.06 (−0.34; 0.48)

*MP_X50, particles trituration; MPB, chewing homogenization; MP_ME_5.6, % material retained in the 5.6 mm sieve; MP_ME_2.8, % material retained in the 2.8 mm sieve*.

*Bold font indicates statistically significant differences*.

**Table 2:** Mixed-effects regression model of the swallowing threshold outcomes (ST- no predefined number of cycles) according to facial pattern (FP), anteroposterior skeletal discrepancy (ASD), sex, and age.

	**Swallowing Threshold test**					
**Outcomes**	**ST_X50 coefficient (95%CI)**	**ST_B coefficient (95% CI)**	**ST_ME_5.6 coefficient (95% CI)**	**ST_ME_2.8 coefficient (95% CI)**	**Cycles coefficient (95% CI)**	**Time coefficient (95% CI)**
**Time**					
3 months	Ref.	Ref.	Ref.	Ref.	Ref.	Ref.
1 year	**0.69 (0.40; 0.98)**	0.07 (−0.75; 0.91)	**0.65 (0.39; 0.92)**	**0.46 (0.22; 0.71)**	**0.61 (0.28; 0.95)**	**0.56 (0.31; 0.82)**
3 years	−0.13 (−0.46; 0.19)	0.07 (−0.09; 0.25)	−0.20 (−0.54; 0.13)	0.15 (−0.11; 0.42)	0.24 (−0.13; 0.62)	0.15 (−0.18; 0.48)
1–3 years	**0.91 (0.68; 1.13)**	**0.15 (0.10; 0.20)**	**0.94 (0.66; 1.23)**	**0.60 (0.29; 0.91)**	**0.56 (0.22; 0.89)**	**0.57 (0.18; 0.97)**
**FP**						
Mesofacial	Ref.	Ref.	Ref.	Ref.	Ref.	Ref.
Brachyfacial	−0.01 (−0.76; 0.73)	**0.04 (0.03; 0.06)**	−0.20 (−0.87; 0.47)	0.57 (−0.20; 1.35)	−0.04 (−0.78; 0.69)	−0.03 (−0.32; 0.26)
Dolichofacial	−0.04 (−1.98; 1.90)	−0.29 (−0.98; 0.40)	0.25 (−2.82; 3.34)	0.69 (−1.18; 2.56)	−0.06 (−1.16; 1.04)	−0.02 (−0.38; 0.32)
**ASD**						
Class I	Ref.	Ref.	Ref.	Ref.	Ref.	Ref.
Class II	**1.17 (0.40; 1.93)**	1.59 (−0.01; 3.20)	**2.97 (0.08; 5.86)**	0.92 (−0.31; 2.17)	–**1.79 (**–**3.27;** –**0.32)**	–**0.75 (**–**1.39;** –**0.11)**
Class III	–**0.59 (**–**0.86;** –**0.32)**	0.02 (−0.01; 0.07)	–**0.60 (**–**1.17;** –**0.04)**	−0.04 (−0.72; 0.63)	1.25 (−0.35; 2.88)	**1.21 (0.04; 2.39)**
**Sex**						
Male	Ref.	Ref.	Ref.	Ref.	Ref.	Ref.
Female	−0.15 (−0.49; 0.19)	−0.04 (−0.22; 0.14)	−0.10 (−0.22; 0.01)	−0.01 (−0.31; 0.27)	0.04 (−0.84; 0.93)	0.04 (−0.52; 0.61)
**Age (years)**	−0.00 (−0.05; 0.04)	−0.13 (−0.52; 0.24)	0.22 (−0.63; 1.07)	0.02 (−0.45; 0.50)	0.82 (−0.26; 1.91)	0.67 (−0.23; 1.58)

*MP_X50, particles trituration; MPB, chewing homogenization; MP_ME_5.6, % material retained in the 5.6 mm sieve; MP_ME_2.8, % material retained in the 2.8 mm sieve*.

*Bold font indicates statistically significant differences*.

**Figure 1:**
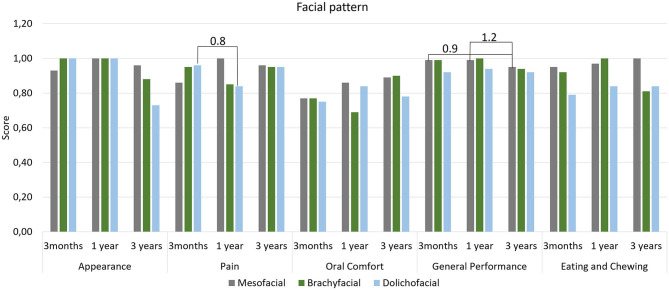
Mean scores of DIDL questionnaire domains according to facial pattern. Periods with large effect size are indicated.

**Figure 2:**
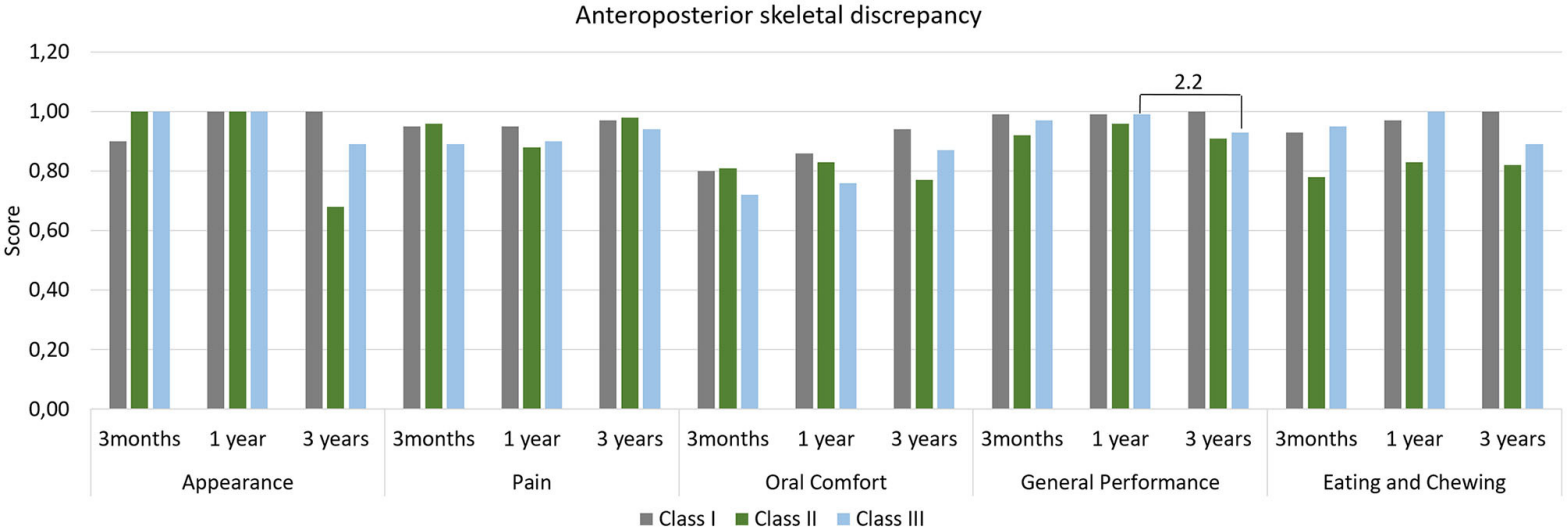
Mean scores of DIDL questionnaire domains according to anteroposterior skeletal discrepancy. Periods with large effect size are indicated.

**Figure 3:**
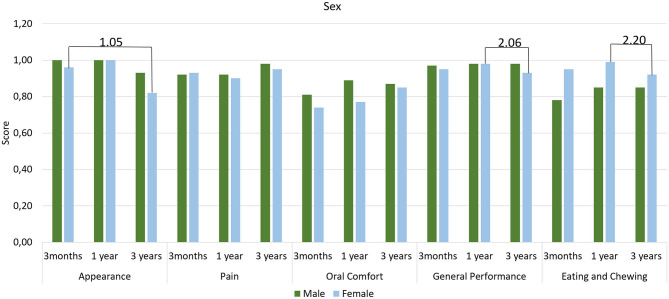
Mean scores of DIDL questionnaire domains according to sex. Periods with large effect size are indicated.

Table 3 lists the coefficients and confidence intervals obtained for OHRQoL domain scores and shows that Brachyfacial and Mesofacial individuals reported differences in the Appearance domain (*p* ≤ 0.01). Dolichofacial and Mesofacial individuals experienced different Oral Comfort (*p* ≤ 0.01), while Class III and Class I individuals experienced a reduction in this same domain (*p* ≤ 0.01). After the 3rd year, age resulted in differences in the Pain (*p* ≤ 0.01), Oral Confort (*p* ≤ 0.01) and General Performance (*p* ≤ 0.01). Figures 1–3 illustrate the changes in DIDL scores over time within each group. The Pain domain scores of dolichofacial individuals reduced by 12.50% between 3 months and year 1 (ES 0.8). For Mesofacial individuals, there was a 4.04% reduction in the average General Performance domain score between 3 months and 3 years and between 1 and 3 years (ES 0.9 and ES 1.2, respectively). Finally, Class III individuals reported a 6.06% reduction in the General Performance domain score between 1 and 3 years old (ES 2.2). Finally, women reported a reduction of 14.58% in the Appearance domain between 3 months and 3 years (ES 1.05), while their General Performance (ES 2.06) and Eating and Chewing (ES 2.20) domain scores reduced by 5.10 and 7.07%, respectively, between 1 and 3 years.

**Table 3:** Mixed-effects regression model of DIDL domain scores according to facial pattern (FP), anteroposterior skeletal discrepancy (ASD), sex, and age.

	**DIDL**				
	**Appearance, coefficient (95%CI)**	**Pain, coefficient (95%CI)**	**Oral comfort, coefficient (95%CI)**	**General performance, coefficient (95%CI)**	**Eating and chewing, coefficient (95%CI)**
**Time**					
3 months	Ref.	Ref.	Ref.	Ref.	Ref.
1 year	*	−0.26 (−0.67; 0.14)	0.20 (−0.11; 0.52)	**1.61 (0.99; 2.22)**	**0.84 (0.63; 1.06)**
3 years	−0.26 (−0.16; 0.11)	**1.63 (0.89; 2.37)**	0.27 (−0.17; 0.73)	−0.12 (−0.38; 0.14)	0.21 (−0.07; 0.49)
1–3 years	*	**0.77 (0.24; 1.31)**	0.14 (−0.31; 0.61)	0.01 (−0.12; 0.16)	**0.78 (0.43; 1.14)**
**FP**					
Mesofacial	Ref.	Ref.	Ref.	Ref.	Ref.
Brachyfacial	**0.49 (0.40; 0.59)**	−0.40 (−1.85; 1.05)	0.05 (−0.57; 0.62)	−0.55 (−1.79; 0.69)	−0.16 (−0.85; 0.52)
Dolichofacial	0.03 (−0.76; 0.15)	2.59 (−1.87; 1.87)	**−0.77 (−1.12; 0.68)**	−0.65 (−2.55; 1.24)	−0.14 (−0.74; 0.45)
**ASD**					
Class I	Ref.	Ref.	Ref.	Ref.	Ref.
Class II	−0.05 (−0.22; 0.11)	*	0.08 (−0.10; 0.27)	*	*
Class III	−0.92 (−3.62; 1.76)	−0.10 (−0.49; 0.29)	**0.49 (0.26; 0.72)**	*	*
**Sex**					
Male	Ref.	Ref.	Ref.	Ref.	Ref.
Female	0.02 (−0.14; 0.19)	−0.06 (−0.38; 0.26)	−0.00 (−0.54; 0.53)	−0.05 (−0.21; 0.11)	−0.32 (−1.40; 0.77)
**Age (years)**	0.00 (−0.014; 0.023)	**0.00 (0.00; 0.01)**	**0.01 (0.00; 0.02)**	**0.00 (0.00; 0.01)**	0.01 (−0.00; 0.02)

*Bold font indicates statistically significant differences*.

* *Variables show collinearity: constant variables*.

The masticatory outcomes at all evaluation periods are listed in the Tables 4, 5 and show that significant differences were observed only for the swallowing threshold tests. Table 5 shows that the ST_X50 values only reduced significantly in male individuals between 3 months and 1 year by 7.83% (*p* ≤ 0.01).

**Table 4:** Masticatory Performance (MP) outcomes (mean ± standard deviation) over time according to facial pattern, anteroposterior skeletal discrepancy and sex (Wilcoxon-paired test).

		**Facial pattern**			**Anteroposterior skeletal discrepancy**			**Sex**	
		**Mesofacial**	**Brachyfacial**	**Dolichofacial**	**Class I**	**Class II**	**Class III**	**Male**	**Female**
MP_X50: (mm)	3 months	4.23 (1.10)	4.78 (1.44)	4.17 (1.26)	4.28 (1.15)	4.20 (1.28)	4.55 (1.36)	3.62 (1.18)	4.72 (1.17)
	1 year	3.94 (1.00)	4.73 (1.22)	3.94 (1.01)	4.42 (1.18)	3.84 (0.96)	4.29 (1.17)	3.54 (0.92)	4.46 (1.08)
	3 years	3.43(1.24)	4.64 (1.15)	3.99 (0.70)	3.92 (1.37)	3.90 (0.83)	4.04 (1.23)	3.72 (0.91)	4.08 (1.23)
MP_B	3 months	3.27 (1.35)	4.77 (2.69)	3.99 (3.87)	3.34 (0.70)	4.16 (4.19)	4.23 (2.57)	3.11 (0.68)	4.36 (3.29)
	1 year	3.53 (2.24)	4.42 (2.06)	3.10 (0.38)	3.48 (1.85)	3.05 (0.39)	4.16 (1.17)	2.86 (0.46)	4.02 (2.07)
	3 years	3.11 (1.00)	4.33(2.85)	3.10 (0.46)	3.13 (1.12)	3.02 (0.54)	3.91 (2.30)	2.99 (0.51)	3.67 (2.00)
MP_ME_5.6: (%)	3 months	26.05 (20.97)	38.38 (28.32)	25.23 (25.54)	25.12 (19.97)	23.63 (17.31)	34.33 (27.01)	17.03 (19.43)	34.17 (22.59)
	1 year	23.39 (19.54)	34.82 (25.22)	17.92 (18.33)	31.26 (18.08)	19.32 (19.02)	25.83 (24.85)	12.15 (14.47)	30.83 (22.05)
	3 years	15.81 (21.07)	34.66 (24.75)	15.63 (10.48)	23.85 (24.66)	15.72 (11.45)	23.00 (23.31)	14.32 (11.70)	24.26 (23.31)
MP_ME_2.8: (%)	3 months	22.81 (8.54)	17.28 (12.97)	21.40 (9.49)	21.45 (10.16)	21.54 (9.11)	19.34 (11.34)	25.63 (8.74)	18.23 (10.18)
	1 year	22.19 (10.70)	16.81 (12.96)	26.17 (7.97)	17.57 (9.26)	25.45 (7.82)	21.61 (13.21)	26.64 (7.20)	19.73 (11.90)
	3 years	24.89 (9.19)	17.23 (10.06)	24.32 (5.51)	22.49 (10.32)	24.13 (5.84)	21.50 (9.83)	25.02 (7.18)	21.35 (9.40)

*Statistically significant differences were not found*.

**Table 5:** Swallowing threshold outcomes (mean ± standard deviation) over time according to facial pattern, anteroposterior skeletal discrepancy, and sex (Wilcoxon-paired test).

		**Facial pattern**			**Anteroposterior skeletal discrepancy**			**Sex**	
		**Mesofacial**	**Brachyfacial**	**Dolichofacial**	**Class I**	**Class II**	**Class III**	**Male**	**Female**
ST_X50: (mm)	3 months	3.81 (0.98)	4.53 (0.98)	3.65 (0.89)	3.72 (1.15)	3.70 (0.98)	4.24 (0.99)	3.32 (0.93)^*^	4.23 (0.97)
	1 year	3.47 (1.33)	4.21 (1.34)	3.49 (0.94)	3.91 (1.29)	3.33 (0.94)	3.85 (1.37)	3.06 (0.72)^*^	3.99 (1.31)
	3 years	3.07 (1.06)	4.34 (1.35)	3.41 (0.60)	3.29 (1.23)	3.39 (0.66)	3.73 (1.29)	2.92 (0.67)	3.78 (1.17)
ST_B	3 months	2.88 (0.93)	4.93 (4.83)	3.12 (1.21)	3.17 (1.78)	3.19 (1.33)	4.05 (4.00)	3.05 (1.74)	3.81 (3.31)
	1 year	3.43 (1.92)^#^	3.68 (2.21)	2.93 (0.61)	3.70 (2.36)^#^	2.97 (0.52)	3.41 (1.87)	2.87 (0.60)^#^	3.55 (1.98)
	3 years	2.64 (0.80)^#^	3.40 (1.99)	2.83 (0.37)	2.70 (0.89)^#^	2.79 (0.44)	3.11 (1.58)	2.52 (0.44)^#^	3.10 (1.34)
ST_ME_5.6: (%)	3 months	18.11 (14.64)	32.34 (19.29)	13.69 (14.04)	17.58 (16.58)	15.40 (15.01)^#^	26.61 (18.63)	11.66 (12.25)^#^	25.25 (18.04)
	1 year	14.88 (24.05)	31.01 (24.85)	12.14 (17.06)	26.87 (29.00)	9.72 (15.88)^#^	21.10 (23.28)	7.15 (11.16)^#^	24.16 (25.28)
	3 years	9.66 (15.06)	26.07 (21.85)	13.75 (15.51)	18.50 (21.67)	9.62 (9.35)	18.04 (20.31)	6.49 (4.90)	20.00 (20.48)
ST_ME_2.8: (%)	3 months	22.88 (7.88)	18.26 (11.39)	24.30 (7.98)	21.94 (8.61)	25.75 (8.61)	19.50 (9.65)	25.76 (8.15)	20.21 (9.35)
	1 year	22.85 (11.53)	20.63 (11.01)^#^	27.50 (10.95)	24.37 (14.65)	27.76 (9.28)	20.69 (10.24)	29.27 (9.44)	21.25 (11.26)
	3 years	25.00 (9.59)	16.33 (10.49)^#^	28.54 (7.46)	21.35 (9.86)	28.94 (6.62)	21.83 (11.55)	26.12 (5.78)	22.65 (11.66)
Time: (sec)	3 months	59.48 (31.03)	56.14 (23.59)	62.98 (19.09)^#^	57.05 (23.28)	62.04 (15.64)	60.83 (30.08)	62.40 (25.07)	59.19 (24.58)
	1 year	60.40 (36.86)	56.95 (22.96)	56.18 (13.24)	47.32 (12.79)	53.82 (12.71)	65.98 (34.50)	55.05 (13.70)	59.22 (30.06)
	3 years	56.37 (14.02)	60.37 (26.13)	47.46 (19.25)^#^	57.53 (24.67)	49.05 (20.49)	56.11 (17.31)	63.97 (18.13)	50.10 (19.59)
Cycles	3 months	69.73 (37.73)	60.31 (23.61)	73.71 (29.36)	70.64 (35.62)	73.33 (28.61)^#^	63.42 (30.35)	79.46 (36.74)	63.07 (27.04)
	1 year	65.71 (37.09)	62.25 (16.28)	56.62 (15.31)	51.78 (14.96)	57.67 (15.50)^#^	69.17 (32.23)	61.83 (14.66)	61.52 (28.96)
	3 years	63.31 (25.93)	67.40 (28.10)	57.08 (18.18)	63.89 (31.82)	60.00 (18.38)	62.94 (23.36)	74.18 (24.31)	56.92 (22.12)

# *Shows statistically significant difference according to Wilcoxon-paired (p = 0.05)*.

* *Shows statistically significant difference according to Wilcoxon-paired test using Bonferroni correction of the P-values (p = 0.0166)*.

*Exact p values found according to the intragroup comparisons: Mesofacial= ST_B (1–3 y, p = 0.023); Brachyfacial = ST_ME_2.8 (1–3 y, p = 0.022); Dolichofacial = Time (3 m−3 y, p = 0.050); Class I = ST_B (1–3 y, p = 0.036); Class II = ST_ME_5.6 (3 m−1 y, p = 0.034), Cycles (3 m−1 y, p = 0.041); **Male** = **ST_X50 (3 m**–**1 y**, ***p* =0.015)**, ST_B (1–3 y, p = 0.028), ST_ME_5.6 (3 m−1 y, p = 0.019)*.

## Discussion

In our study population, a robust regression analysis indicated important changes in the mean values of the MF and OHRQoL variables over the 3-year follow-up period. Brachyfacial individuals continue to show worse food homogenization than Meso- and Dolichofacial individuals in year 3 for both masticatory function tests. Meanwhile, food trituration abilities of Class II and Class III individuals deteriorated slightly, while their masticatory efficiency (ME_5.6) improved and Class II individuals needed fewer masticatory cycles after 3 years. The improvements in masticatory efficiency are clinically insignificant for Class III individuals, but fairly large for Class II individuals. Finally, the various OHRQoL, domains continue to be influenced by both FP, ASD and age, in the same period.

Mesofacial individuals showed improvements over time after transition to IMO, as indicated by a reduction in their B values between the 1st and the 3rd year that reflects an improvement in particle homogenization. This continuous improvement can be explained by the vertical growth (1) and balanced facial musculature activity, added to the continued long-term improvement in retention and stability promoted by IMO. Conversely, Brachyfacial individuals showed changes in the monitored parameters both over time and in relation to Mesofacial individuals. The ME_2.8 percentage in these individuals reduced by 20.84% between 1st and the 3rd year of function from 20.63 to 16.33%, showing that the medium-term use of IMO did not improve the fine particle trituration capacity of brachyfacial individuals. This worsening of the trituration ability can be attributed to reduced growth and height of the lower third of the face (8), which lowers the amplitude of mandibular movement during chewing, resulting in reduced mobility of the bolus in the oral cavity. Thus, the mandibular kinematics of edentulous brachyfacial individuals may have contributed to a smaller number of chewed particles that reached the 2.8 mm sieve. In this sense, these results support the idea that a larger intra-oral space favors more efficient breakdown of food particles during chewing (10).

In addition, Brachyfacial individuals also obtained less homogenous food boluses than Mesofacial individuals in the third year for both masticatory tests. This shows that the rehabilitation with IMO did not promote the expected improvement of all outcomes related to masticatory capacity, the most sensitive being the particle homogenization, followed by ME_2.8, as both outcomes started to worsen in the third year compared with the reference group (Mesofacial individuals). For Dolichofacial individuals, the masticatory cycles needed to complete the ST test reduced over time, which can be explained by the greater intraoral space that facilitates handling the food bolus and pulverization of the particles, reducing the time needed to perform the masticatory cycles (10).

In terms of ASD, only Class I individuals still showed changes in some masticatory variables in the 3rd year, as their particle homogenization capabilities improved between the 1st and 3rd year. As the homogenization capabilities of Mesofacial individuals also improved, it seems likely that the improved retention and stability provided by IMO, added to the fact that these patients feel safer when chewing, are factors that contribute to medium-term improvements in particle homogenization capabilities. Furthermore, Classes II and III showed differences in relation to Class I for various ST parameters, including X50, ME_5.6, and cycle time. Class II individuals showed slightly worse average particle crushing capacity in the 3rd year, however, the ability to triturate coarse particles, and the time and number of cycles simultaneously showed improvements. The slightly worse crushing ability of Classes II and III may be related to the reduced time taken for chewing, as this can directly interfere with particle crushing (25, 26). In this context, the study by Van der Bilt (27) showed that subjects with good masticatory function, do not necessarily swallow food after a smaller number of cycles, as the ST is directly influenced by the physiology of the individual aside from the social context wherein the individual is included, as the social context can induce the patient to chew more quickly. In addition, the mandibular protrusion in Class III and maxillary protrusion in Class II individuals may also be responsible for these differences in MF in the 3rd year of IMO function.

It is well-known that sex can influence MF even after rehabilitation with IMO. In our study population, males still showed changes in particle homogenization capacity after 3 years. Hatch et al. (28) found that sex was the factor that most influenced the bite force, mainly due to the larger thickness of the masseter which is the main contributing factor to a greater bite force. Thus, the improvement in the particle homogenization for male individuals may be related to development of the masseter during medium-term IMO use. In this context, the literature showed that the bite force and the MF continue to improve over 3 years of IMO use (29), and show that after treatment with implants there is a long-term neuromuscular adaptation, and report an increase in myodynamic parameters and electromyography, approximating the values of dentate individuals (30), corroborating our results.

Presently, little is known about the influence of facial morphology on OHRQoL and patient satisfaction. Faot et al. (10) observed that treatment with IMO positively impacts OHRQoL after 3 months of rehabilitation, especially in the Oral Comfort domain. In the present study, however it was observed that FP can still influence OHRQoL medium-term, since individuals with Dolichofacial and Brachyfacial features reported distinct scores in various domains in the 3rd year, where Dolichofacial individuals reported a worse score (11%) in the Oral Comfort domain and Brachyfacial individuals reported a worse score (8%) in the Appearance domain compared to the reference group (Mesofacial individuals). In terms of ASD, only Class III still shows significantly lower scores (7%) than Class I individuals in the Oral Comfort domain after 3 years. While Faot et al. (10) found no differences in subjective perception in both types of PF and ASD 3 months after loading the IMO our results indicate that on the medium- to long-term, OHRQoL is influenced by different facial patterns and anteroposterior discrepancies. Moreover, age influenced the OHRQoL regardless of facial patterns, mainly in the Pain, Oral Comfort and General Performance domains. These results are in accordance with Schuster et al. (31) wherein the authors observed that individuals aged ≥65 years reported worse domain scores than individuals aged <65 years, reflecting a decrease in OHRQoL with increasing age. The effect size analysis reveals that Mesofacial and Class III individuals reported a reduction in the General Performance domain in the 3rd year compared to the first. However, in general facial patterns had limited influence on the OHRQoL outcomes, as most domains maintained an average score > 0.7, reflecting overall satisfaction with the treatment.

The limitations of this study include the lack myographs, especially of the masseter, and bite force measurements, as these directly influence MF. Another limitation relates to the lack of studies available for direct comparison of these results, due to the scarcity of studies in literature assessing medium-term effects of MF and OHRQoL as a function of facial morphology (FP and ASD). The present study assessed a relatively small number of patients (*n* = 40) given the amount of parameters needed to describe these relationships. Extrapolation of our results to different populations should be done with caution. More studies with larger sample sizes, more diverse sample populations, and assessment of more parameters related to mastication are required to understand the medium-term relationships between oral health-related quality of life, mastication, and facial morphology.

## Conclusion

The masticatory performance and oral health-related quality of life parameters of implant mandibular overdenture users change over time as a function of facial pattern, anteroposterior skeletal discrepancies, and sex. In our study population, the differences in facial morphology continued to influence the masticatory function and oral health-related quality of life in the 3rd year of implant mandibular overdenture function, and age can influence some OHRQoL domains; brachyfacial individuals benefited least from rehabilitation with IMO, as several masticatory outcomes deteriorated, such as particle homogenization and masticatory efficiency (ME_2.8).

## Data Availability Statement

The original contributions presented in the study are included in the article and further inquiries can be directed to the corresponding author.

## Ethics Statement

The studies involving human participants were reviewed and approved by Research Ethics Committee of the Faculty of Dentistry UFPel, protocol (No. 69/2013). The patients/participants provided their written informed consent to participate in this study.

## Author Contributions

FF, AP, and AM: conceptualization and project administration. AP, AS, RM-M, AM, LP, and OC-J: methodology, patient's treatment, and clinical follow-ups. AP, AS, RM-M, and AM: writing—original draft preparation, software, data curation, and visualization. FF, LP, OC-J, and AD: investigation. FF, LP, and OC-J: formal analysis, resources, and supervision. FF, AP, AS, RM-M, and AD: writing—review and editing. All authors contributed to the article and approved the submitted version.

## Conflict of Interest

The authors declare that the research was conducted in the absence of any commercial or financial relationships that could be construed as a potential conflict of interest.
